# Simulation of Flow Field and Experimental Study on the Electric Discharge Machining of Small Holes in Renewable Dielectrics

**DOI:** 10.3390/mi16070767

**Published:** 2025-06-29

**Authors:** Ruili Wang, Yangjing Zhao, Binghui Dong, Shuo Sun, Na Xiao, Wuyi Ming

**Affiliations:** 1 Department of Engineering, Huanghe University of Science and Technology, Zhengzhou 450008, China; 201309111@hhstu.edu.cn (R.W.); 201608156@hhstu.edu.cn (N.X.); 2 Mechanical and Electrical Engineering Institute, Zhengzhou University of Light Industry, Zhengzhou 450002, China; zhaoyj0529@163.com (Y.Z.); dongbh1027@163.com (B.D.); sunshuo202304@163.com (S.S.); 3 Guangdong HUST Industrial Technology Research Institute, Huazhong University of Science and Technology, Dongguan 523808, China

**Keywords:** electrical discharge machining, EDM hole machining, renewable dielectric, discrete phase model

## Abstract

Vegetable oil is regarded as a medium that can replace kerosene in electrical discharge machining (EDM) hole processing due to its renewability and environmental friendliness. Meanwhile, numerical simulation serves as an effective means to study the behavior of the gap flow field during EDM processing. Based on this, this study explored the influence of hole size and different vegetable oil dielectrics (sunflower seed oil, canola oil, and soybean oil) on the movement of electro-corrosion residues in the processing gap. The simulation results demonstrate that the viscosity of the oil affects the escape rate of the particles. In holes of 1 mm and 4 mm of size, the escape rate of canola oil at any time period is superior to that of sunflower seed oil and soybean oil. In a 1 mm hole, its average escape rate reached 19.683%, which was 0.24% and 0.19% higher than that of sunflower seed oil and soybean oil, respectively. Subsequently, experiments were conducted in combination with the simulation results to explore the influence of current, pulse width, and pulse interval on hole processing. This further confirmed the application potential of vegetable oil in electrical discharge micro-hole processing and provided theoretical support and experimental basis for optimizing the green manufacturing process.

## 1. Introduction

The origin of electric discharge machining (EDM) can be traced back to the 1940’s. In 1943, the Soviet scholars, the Lazarenkovs couple, in their study of the mechanism of corrosion damage to switch contacts due to spark discharges, discovered that the transient high temperatures generated by electric sparks could cause localized metal melting, vaporization, and removal. Based on this discovery, they put forward the idea of using the principle of galvanic corrosion for material removal and applied it to manufacturing, inventing the EDM method [1]. In this machining process, EDM removes the material through the galvanic corrosion effect produced by the pulse discharge between the electrode and the workpiece, realizing precision machining of the workpiece’s size, shape and surface quality [2]. In the actual machining process, the workpiece and the tool are connected to the poles of the pulsed power supply and submerged in a liquid dielectric. Under the regulation of the automatic feeding device, a certain gap is maintained between the tool electrode and the workpiece. When a certain voltage is applied, a pulsed spark discharge occurs in the gap, leaving a tiny crater on the surface of the workpiece after each discharge. Under the effect of continuous discharge at high frequency, these craters gradually accumulate, and the shape of the tool electrode is copied onto the workpiece. By precisely controlling the pulse frequency, current amplitude, pulse width, and other parameters, the machining process can be effectively controlled to meet the requirements of different workpieces in terms of size, shape, and surface quality [3]. To gain a deeper understanding of the material removal mechanism during the discharge process, Alshaer et al. [4] utilized a meshless, smoothed-particle hydrodynamics multiphase model to simulate EDM processes on Ti–6Al–4V and AISI304 steel, accurately predicting crater size, aspect ratio, and material removal rate with an error of only 2%–22%, significantly outperforming traditional finite element models. Similarly, Papazoglou et al. [5] numerically studied how plasma channel radius evolution affects energy absorption, flushing efficiency, and crater shape. They found slower channel growth reduces energy absorption but improves material removal, while a higher power-law exponent produces narrower, deeper craters.

The processing of small holes by EDM has irreplaceable importance in the field of precision manufacturing. Through the principle of high-frequency discharge, it can realize 0.1 mm level micro-hole processing on hard-to-cut materials such as super-hard alloys, ceramics, etc., which breaks through the physical limitations of traditional drilling, especially in the key scenarios of the aero-engine holes. Dong et al. [6] experimentally studied surface quality improvement of Be-Cu alloys using micro-EDM with multi-diameter electrodes and different dielectrics. They used a segmented electrode design, applying water as the dielectric for small diameters and kerosene for large diameters. Results showed this approach improved micropore surface morphology, achieving an Ra as low as 0.35 μm. In addition, in order to further expand the application of EDM in the machining of complex shapes, Zou et al. [7] studied drilling 2 mm shaped heterotypic holes using a circular cross-section electrode in EDM. They found that reducing peak current, pulse width, and pulse interval greatly improved surface roughness and accuracy. However, smaller machining sizes and complex shapes increased discharge uncertainty, causing more short circuits and arc discharges, leading to unstable discharges, lower accuracy, and higher electrode wear. To address the uncertainties in electrode wear and the discharge process, Arun et al. [8] developed a numerical model for single discharges in micro-EDM, predicting the crater size on copper electrodes. The error in electrode wear rate and erosion depth was approximately 20%. Results showed that, as the crater overlap ratio increased from 0% to 50%, the electrode wear rate decreased from 20.4% to 8%.

In addition, the tool and workpiece are always immersed in a dielectric during the EDM process. The dielectric not only acts as an insulating medium between the poles, but also compresses the discharge channel and cools the machining area during the discharge. Moreover, the dielectric is responsible for flushing and discharging the debris generated during machining to avoid its accumulation in the discharge gap, thus guaranteeing the stability of subsequent machining [9]. Traditionally, kerosene and other hydrocarbon oils are commonly adopted as dielectrics for EDM [10,11]. However, under high temperature, such oils are prone to thermal cracking and decomposition reactions, releasing large amounts of hazardous fumes and aerosols, which pose a threat to the environment and the health of the operators [12]. A study by Mathew et al. [13] found that a wide range of toxic exhausts are generated during the EDM process. Taking kerosene as an example, it can generate as many as 53 gases, including 13 light gases below C6 and 40 heavy gases above C6, such as benzene, toluene, and other substances that are harmful to human body. These gases not only pollute the environment, but also may cause health problems such as skin diseases [14]. Tönshoff et al. [15] reported that hydrocarbon dielectrics release harmful substances such as aliphatic hydrocarbons, aerosols, benzene, and fine dust during EDM. Sivapirakasam et al. [16] observed that aerosol particles generated in EDM were spherical nanoparticles, which were potentially a health risk. Sai Ram et al. [17] stated that these aerosols and toxic gases could cause serious occupational and environmental problems. Therefore, the high energy consumption, dielectric waste disposal, and toxic emissions in EDM processes have attracted much attention, and researchers have begun to explore alternative solutions.

Advanced manufacturing processes, such as laser machining and ultrasonic-assisted processing, can significantly enhance processing performance by improving machining accuracy and efficiency [18]. To reduce the environmental and health impacts of conventional dielectrics while preserving insulation and corrosion resistance, researchers are developing green dielectrics. Bio-based dielectrics, renewable, biodegradable, and low in toxicity, are a promising alternative to kerosene. Valaki et al. [19] used palm oil instead of hydrocarbon oil as a dielectric, increasing MRR by 38% with similar surface roughness (SR) and hardness. Ahmad et al. [20] observed a 158.56% higher MRR with palm oil compared to kerosene, though the electrode wear rate (EWR) was slightly higher than SR. These studies indicate that vegetable-oil-based dielectrics can replace mineral oils and, in some cases, offer better processing performance. Valaki et al. [21] further showed that jatropha-oil-based dielectrics offer better MRR and surface hardness and lower SR than kerosene. Additionally, waste vegetable oil (WVO) and its blend (BUVO) were tested as dielectrics, with WVO achieving higher MRR than BUVO and hydrocarbon oils [22]. Despite performance variations among vegetable oils in EDM, their environmental benefits and processing advantages are making them strong competitors to mineral oils.

This study investigates the feasibility of using renewable dielectrics for small hole machining in EDM through flow field simulation and experiments. A 3D fluid simulation system based on the discrete phase model (DPM) is built to analyze the effects of dielectric type and hole diameter on particle motion using sunflower seed, soybean, and canola oils. Based on the simulation, experiments on SKD11 mold steel explore how current, hole diameter, depth, pulse width, and duty cycle affect machining performance. The study offers theoretical and experimental support for understanding EDM parameter effects on particle behavior, aiding in process optimization and reducing trial-and-error costs.

## 2. Simulation Model of Flow Field for Small-Hole Machining

### 2.1. Simulation Methods

In this study, a DPM model is adopted to simulate the flow field dynamics of the eroded particle population in the EDM gap. In order to improve the computational efficiency and focus on the core physical mechanism, the model is constructed by emphasizing the dominant hydrodynamic mechanism and treating the nonlinear complex factors such as the electromagnetic coupling effect and blast shock wave and thermos–fluid–solid coupling as higher-order variables for the time being. Based on the typical electric discharge machining environment, the modeling follows the following core boundary conditions:

(1) The medium property is set as an incompressible pure liquid dielectric;

(2) The source of the discrete phase is defined as the continuous spray of EDM particles on the workpiece surface;

(3) The processing interface has a spatial and temporal uniformity of particle spraying dynamic characteristics;

(4) A particle kinematics model is constructed so that it is dominated by the flow field control equations, solved by the velocity field through the Navier–Stokes equations, and combined by the initial momentum parameters of the particles with the wall collision boundary conditions to realize the quantitative analysis of the migration trajectory of the etchers.

The proposed model makes key assumptions based on actual working conditions and provides a controllable numerical experimental platform for revealing the microscale-flow field–particle interaction mechanism.

### 2.2. Simulation Models

Fluid mechanics, as a basic discipline to study the fluid motion characteristics and its interaction law with solid interfaces, has a three-pillar system of statics, kinematics, and dynamics that constitutes the theoretical foundation of the numerical simulation of fluids [23]. Based on this theoretical framework, the accuracy of the numerical simulation of the microscale flow field can be significantly improved by constructing a mathematical–physical model of the Navier–Stokes system of equations. Within the microgap of EDM, the liquid dielectric, as a typical incompressible fluid, has a physical property of constant density that directly reflects the constraints of the law of mass conservation, from which the continuity Equation (1) can be established in the case of two-dimensional flow [24]:

(1) $$\frac{\partial u}{\partial x}+\frac{\partial v}{\partial y}=0$$

where u and v are the velocities of the fluid along the x and y directions, respectively.

At the same time, the dielectric motion in the interstitial flow field follows the law of conservation of momentum, and its momentum Equations (2) and (3) [24] are as follows:

(2) $$\frac{\partial }{\partial t}\left(pu_{i}\right)+\frac{\partial }{\partial t}\left(pu_{i}u_{j}\right)=-\frac{\partial p}{\partial x_{i}}+\frac{\partial \tau_{ij}}{\partial x_{j}}+pg_{i}+F_{i}$$

(3) $$\tau_{ij}=\left[\mu \left(\frac{\partial u_{i}}{\partial x_{j}}+\frac{\partial u_{j}}{\partial x_{i}}\right)\right]-\frac{2}{3}\mu \frac{\partial u_{l}}{\partial x_{l}}\delta_{ij}$$

where p is the static pressure, $\tau_{ij}$ is the stress tensor, $g_{i}$ is the gravitational volume force in the direction, and $F_{i}$ is the external volume force in the i direction.

The axial and radial momentum conservation equations for a two-dimensional axisymmetric geometry are shown in the following Equations (4) and (5):

(4) $$\begin{array}{l} \frac{\partial }{\partial t}\left(pu\right)+\frac{1}{r}\frac{\partial }{\partial x}\left(rpuu\right)+\frac{1}{r}(rpvu)\\ =-\frac{\partial p}{\partial x}+\frac{1}{r}\left[r\mu \left(\frac{\partial v}{\partial x}+\frac{\partial u}{\partial r}\right)\right]\\ +\frac{1}{r}\frac{\partial }{\partial r}\left\{r\mu \left[\frac{2\partial v}{\partial x}-\frac{2}{3}\left(\nabla \bar{v}\right)\right]\right\}-2\mu \frac{u}{r^{2}}+\frac{2}{3}\frac{u}{r}\left(\nabla \bar{v}\right)+p\frac{w^{2}}{r}+F_{x} \end{array}$$

(5) $$\begin{array}{l} \frac{\partial }{\partial t}\left(pv\right)+\frac{1}{r}\frac{\partial }{\partial x}\left(rpuv\right)+\frac{1}{r}(rpvv)\\ =-\frac{\partial p}{\partial r}+\frac{1}{r}\left[r\mu \left(\frac{\partial v}{\partial x}+\frac{\partial u}{\partial r}\right)\right]\\ +\frac{1}{r}\frac{\partial }{\partial r}\left\{r\mu \left[\frac{2\partial v}{\partial x}-\frac{2}{3}\left(\nabla \bar{v}\right)\right]\right\}-2\mu \frac{u}{r^{2}}+\frac{2}{3}\frac{u}{r}\left(\nabla \bar{v}\right)+p\frac{w^{2}}{r}+F_{r} \end{array}$$

where $\nabla \bar{v}=\frac{\partial u}{\partial x}+\frac{\partial v}{\partial r}+\frac{v}{r}$ and w are the vortex flow rate.

When studying the kinetic behavior of a two-dimensional axisymmetric incompressible fluid in which the effect of heat transfer is neglected, the kinematic properties and kinetic mechanisms can be described by coupling the above equations.

In the three-dimensional case, the continuity Equations (6)–(9) of the fluid and the Navier–Stokes Equations are listed as below [25]:

(6) $$\frac{\partial u}{\partial x}+\frac{\partial v}{\partial y}+\frac{\partial w}{\partial z}=0$$

(7) $$\rho \left(\frac{\partial u}{\partial t}+u\frac{\partial u}{\partial x}+v\frac{\partial u}{\partial y}+w\frac{\partial u}{\partial z}\right)=X-\frac{\partial p}{\partial x}+\mu \left(\frac{\partial^{2}u}{\partial t^{2}}+\frac{\partial^{2}u}{\partial y^{2}}+\frac{\partial^{2}u}{\partial z^{2}}\right)$$

(8) $$\rho \left(\frac{\partial v}{\partial t}+u\frac{\partial v}{\partial x}+v\frac{\partial v}{\partial y}+w\frac{\partial v}{\partial z}\right)=Y-\frac{\partial p}{\partial y}+\mu \left(\frac{\partial^{2}v}{\partial t^{2}}+\frac{\partial^{2}v}{\partial y^{2}}+\frac{\partial^{2}v}{\partial z^{2}}\right)$$

(9) $$\rho \left(\frac{\partial w}{\partial t}+u\frac{\partial w}{\partial x}+v\frac{\partial w}{\partial y}+w\frac{\partial w}{\partial z}\right)=Z-\frac{\partial p}{\partial x}+\mu \left(\frac{\partial^{2}w}{\partial t^{2}}+\frac{\partial^{2}w}{\partial y^{2}}+\frac{\partial^{2}w}{\partial z^{2}}\right)$$

where, X, Y, and Z is the volume force in three directions, u, v, and w is the velocity component in three directions, ρ is the liquid density, μ is the coefficient of viscosity, p is the pressure per unit volume.

### 2.3. Geometrical Modeling and Meshing

This study adopts a modular modeling strategy and relies on the ANSYS integrated simulation platform to construct a numerical experiment system for the EDM flow field. The technical route follows the progressive process of 3D geometric modeling, computational domain discrete topology construction, and multi-physics field solver integration. Firstly, it was based on the Space Claim parametric modeling module to complete the precise reconstruction of the micro gap geometric architecture and, then, through the ICEM CFD high-level preprocessor to implement the computational domain discrete topology construction, the tool’s unique hexahedral-dominant-structured mesh and tetrahedral/polyhedral hybrid generation algorithm, which can meet the requirements for multi-physics field coupling analysis of the EDM flow field. The tool’s unique hybrid generation algorithm of hexahedral-dominated-structured mesh and tetrahedral/polyhedral mesh can meet the mesh quality requirement of the Jacobi coefficient >0.3 for the multi-physics field coupling analysis. In the field of numerical simulation, the quality of the discretized mesh directly determines the convergence of the numerical solution and the computational efficiency. In this study, we establish a framework for mesh sensitivity analysis and adopt the unstructured mesh with a feature size of 0.2 mm to achieve the Pareto optimization of the resolution of the flow boundary layer and the consumption of computational resources, which is verified by the orthogonality test and the cell distortion validation. After the orthogonality test and the verification of cell warping to confirm that the grid quality is up to the standard, the Fluent solver is applied to construct the SIMPLE algorithm system of pressure–velocity coupling to realize the analysis of multiphase flow dynamics in the flow field of dielectrics. This modeling method ensures that key physical phenomena such as vortex separation and secondary flow are captured while the computational scale is controlled within the carrying range of the HPC cluster [26].

**Figure 1:**
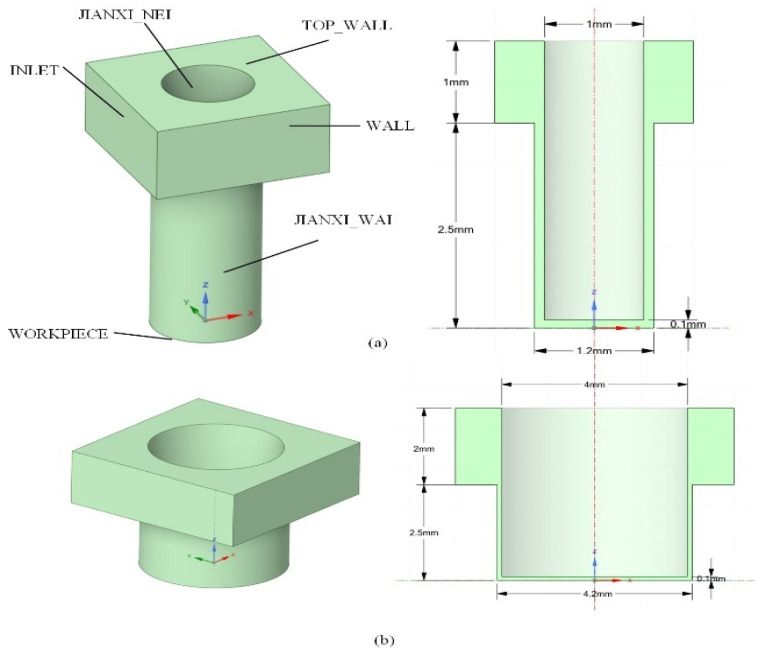
Geometric model of the interelectrode gap flow field: (**a**) diameter = 1 mm; (**b**) diameter = 4 mm.

Based on the actual flushing path characteristics of EDM, a three-dimensional gap flow field numerical model with a physical mapping relationship is constructed (Figure 1), whose free flow field dimensions of the 1.2 mm and 4.2 mm apertures are 3 mm × 3 mm × 1 mm and 6 mm × 6 mm × 2 mm, the heights of the 1.2 mm and 4.2 mm apertures are 2.5 mm, and the gaps of the apertures and the electrodes are all 0.1 mm. The dimensions of the etching interface of the workpieces with the 1.2 mm and 4.2 mm apertures are 1.2 mm and 4.2 mm, and the dimensions of the corresponding electrodes for 1.2 mm and 4.2 mm apertures are 1 mm and 4 mm, respectively. The boundary condition system strictly follows the geometric constraints and flow characteristics of the machining system: the velocity inlet (INLET) and pressure outlet (OUTLET) are set along the Z-axis, corresponding to the supply and return ends of the dielectric, respectively; the core area of the discharge is defined as the electrode-workpiece opposing structure, in which the workpiece etching interface is named as WORKPIECE, and the electrode discharging endface is defined as ELECTRODE; for the complex geometric characteristics of the gap flow channel, the lateral cylindrical surface of the electrode and the annular gap of the hole wall are parameterized as JIANXI_NEI (inner gap boundary) and JIANXI_WAI (outer gap boundary), respectively; the top free liquid surface adopts TOP_WALL to characterize the open flow boundary, and the rest of the lateral wall and the fixed contact surface of the process system are uniformly defined as no slip solid wall (WALL). This boundary condition system realizes the full-scale equivalence between the actual processing geometrical parameters (e.g., gap dimensions, electrode diameters, etc.) and the computational domain through the spatial topological mapping, which establishes an accurate geometrical foundation for the simulation of the flow field dynamics.

### 2.4. Model Parameter Setting

The flow field simulation is mainly designed to investigate the effect of the hole diameter size on the discharge of the erosion products in the plus discharge gap and the performance of different renewable dielectrics in different hole diameters. The settings for experimental levels are shown in Table 1, in which there are three levels of renewable dielectrics, two levels of hole diameters, and one level of processing depth. The three renewable dielectrics selected were sunflower seed oil, soybean oil, and canola oil, and Table 2 shows the physical properties of these three green dielectrics compared to kerosene. In this study, a one-way experiment was adopted, where only one independent variable is varied at a time while keeping all other conditions constant to investigate the distribution of particles under different parameters, the residence time of particles in the gap, and the state of particles at different moments.

In this study, a cross-scale fluid–solid coupling numerical experiment system for small-hole machining of a copper electrode (Φ1/4 mm)-stainless steel SKD11 workpiece is constructed, and a transient multi-physical field coupling numerical model is adopted to accurately reproduce the dynamics of the dielectric flow field in the micrometer-scale discharge gap (typical value of 5–50μm). The model realizes the synergistic simulation of the transient flow field evolution between the electrodes and the workpieces and the turbulence effect on the free liquid surface by means of the fluid–solid coupling algorithm in the submerged machining environment. In particular, it should be pointed out that, for the microscopic scale of the discharge gap and the in situ observation difficulties caused by the dynamic fluctuations of the machining process, this study introduces the dynamic equilibrium equation of the electric discharge machining gap (Equation (10)) [27], which is a theoretical model that provides physical constraints for the numerical simulation through the dynamic correlation of the discharge energy, the machining efficiency, and the dielectric property.

(10) $$\delta_{0}=K_{u}u_{i}+K_{R}W_{M}^{0.4}+\delta_{m}$$

where, $\delta_{0}$ for the discharge gap (refers to the single-sided discharge gap), $u_{i}$ for the open-circuit voltage, $K_{u}$ for the coefficient related to the dielectric strength of the working fluid, $K_{R}$ for the constant related to the material of the workpiece (workpiece for the steel value of 250), $W_{M}$ for the single pulse discharge energy (J), and $\delta_{m}$ for the consideration of the effects of thermal expansion and contraction, vibration and other gaps (generally 2–3 μm).

According to the machining conditions, the discharge gap is 0.1 mm, the gravity is 9.81 m/s^2^ along the Z-axis in the positive direction, the velocity of the oil rush is 2 m/s, and the Reynolds number of the flow field can be obtained from Equation (11),

(11) Re=ρvLη

where ρ is the density of the fluid, v is the flow rate, L is the characteristic length, η and is the viscous coefficient of the fluid.

**Figure 2:**
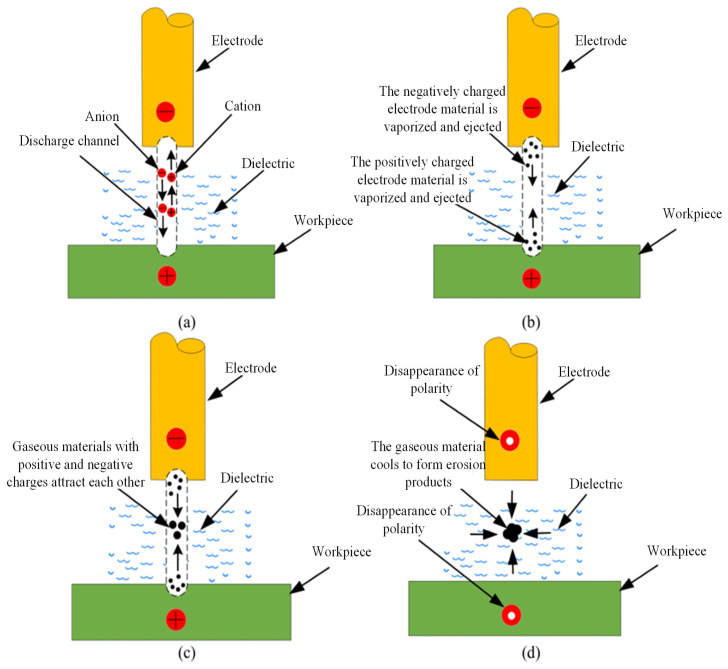
Schematic diagram of the microscopic process of EDM. (**a**) A discharge channel is formed between electrodes by applying discharge pulses; (**b**) electrode surface material melts and vaporizes; (**c**) eroded material accumulates within the discharge channel; (**d**) vaporized products converge and form removal debris.

In this study, a discrete-phase multi-physics field coupled numerical model parameterization configuration strategy is constructed based on the EDM etching particle dynamics’ characteristics, whose EDM microprocess schematic is shown in Figure 2. For the physical properties of the discharge etching product, the discrete-phase material parameter was set as SKD11 steel substrate, the surface-type (surface) particle injection source definition mode was adopted, and the WORKPIECE machining interface was set as the master control area for particle generation. The particle size distribution was parametrically described by the Rosin–Rammler statistical model, which accurately characterizes the polydispersity of micron-sized etched particles in actual processing by setting the distribution index n = 3.5. The spray kinetic parameters were adopted in the normal vector spray mode to ensure that the initial momentum distribution of the particles was physically consistent with the direction of the discharge explosion force.

In the modeling of the discrete phase boundary interaction mechanism, a multi-scale coupling strategy was adopted: OUTLET, WALL, and TOP_WALL were set as escape boundaries to realize the dynamic removal mechanism of the etching particles at the boundary of the flow field. And for the WORKPIECE, ELECTRODE, JIANXI_NEI, and JIANXI_WAI key process interfaces, they were described by momentum conservation equations JIANXI_NEI (inner gap) and JIANXI_WAI (outer gap) and other key process interfaces. The elastic reflection (reflect) boundary conditions were used to describe the collision energy transfer process at the particle–solid interface through the momentum conservation equation. The boundary system effectively reproduced the migration-collision-agglomeration kinetic behavior of micron-sized particles in a confined flow field.

At the solver configuration level, the pressure–velocity coupling is handled using the SIMPLE (Semi-Implicit Method for Pressure-Linked Equations) algorithm to construct the solution framework for the flow field control equations. To enhance the numerical stability of multiphase flow simulations, spatial derivative accuracy is improved by applying the Least Squares Cell-Based (LSCB) gradient reconstruction method. The momentum transport equations are discretized using the Second-Order Upwind Scheme, whose accuracy is approximately two to five times higher than that of dissipative and Roe schemes. This high-order discretization strategy enables the coupled solution of vorticity transport and particle trajectory tracking within the flow field. The specific numerical configuration parameters are detailed in the experimental matrix presented in Table 3.

At the solver configuration level, the SIMPLE algorithm with pressure–velocity coupling was utilized to construct a framework for solving the flow field control equations. In order to improve the numerical stability of multiphase flow, the gradient reconstruction adopted the Least Squares Cell-Based algorithm to optimize the accuracy of spatial derivative calculation, and the discretization format of the momentum transport equation selected the Second-Order Upwind Scheme, whose accuracy is approximately two to five times higher than that of dissipative and Roe schemes [28]. The higher-order discretization strategy realizes the synergistic solution of vortex transport and particle trajectory tracking in the flow field, and the specific computational parameter configurations are shown in the numerical experiment matrix in Table 3.

## 3. Simulation Results and Analysis

The time step of the solution is 0.01 s, the total number of time steps is 100, auto-saving is performed every 10 steps, and the maximum number of iterations for each time step is 10 times. The ratio of particle escape and the size distribution at different moments are calculated, and the results of the simulation experiments are shown in Table 4, where Sso is sunflower seed oil, So is soybean oil, and Co is canola oil for the convenience of recording.

### 3.1. Effect of Hole Size

**Figure 3:**
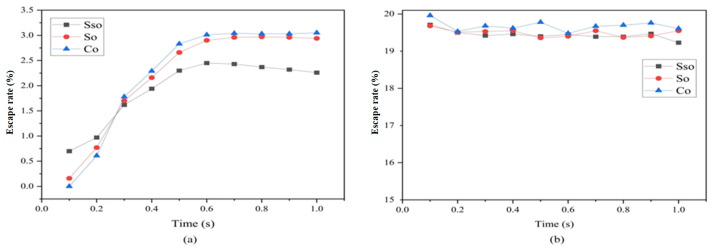
Particle escape rates within 1 s for different small-hole diameters: (**a**) diameter = 1 mm; (**b**) diameter = 4 mm.

Regardless of the renewable dielectric adopted, the escape rate of particles in a 4 mm diameter hole is much higher than that in a 1 mm diameter hole, with the smaller hole size limiting the escape of particles. Figure 3 demonstrates the variation of the escape rate of particles with time when using sunflower seed oil, soybean oil, and canola oil renewable dielectrics in 1 s for machining 1 mm and 4 mm holes. The escape rate of particles within 1 s fluctuates more when the hole with a diameter of 4 mm is processed, but the general trend is decreasing, with the escape rate of particles accounting for the overall highest level at 0.1 s, which reaches the highest value of 19.96% when the renewable dielectric is canola oil. However, when the diameter of the processed holes is reduced to 1 mm, the processing situation shows a completely different situation. In this situation, the escape of particles is in an increasing trend overall, and the escape rate of particles is the lowest at 0.1 s, at which time the best escape rate is achieved in sunflower seeds, with a value of 0.70%. When the renewable dielectric is canola oil, the escape rate of 0 occurs. And, with the increase in time, the particles have the fastest growth in escape rate in sunflower seed oil and remain at the highest level, which is dominant initially and ends up at the lowest level. It is worth noting that the escape rate of particles in a 4 mm diameter hole was about 9.5 times higher than in a 1 mm diameter hole.

**Figure 4:**
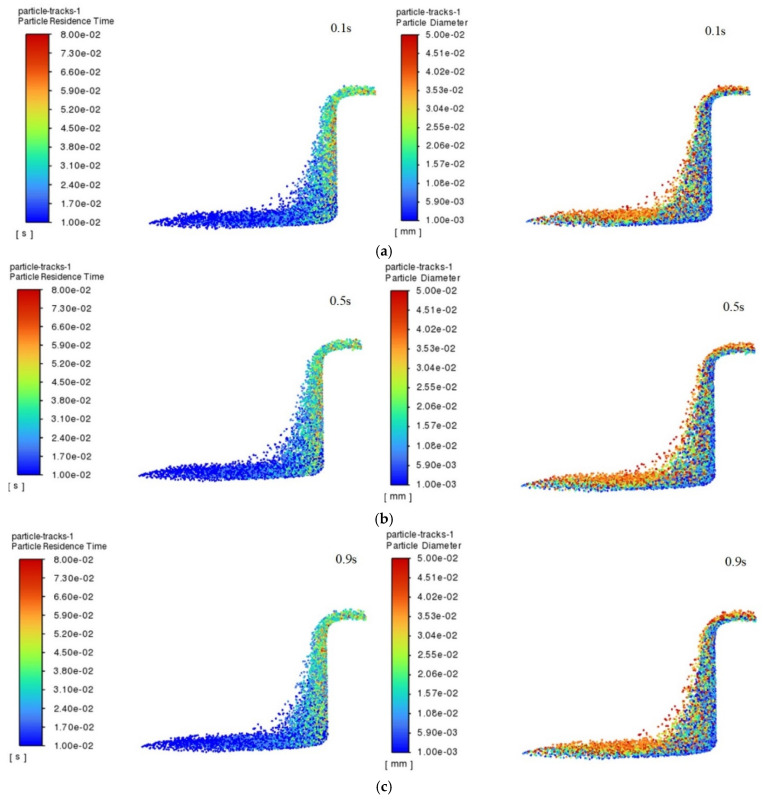
Diameter of the hole was 4 mm, the renewable dielectric was canola oil, and the particle residence time and particle size distributions in 0.1~0.9 s: (**a**) 0.1 s; (**b**) 0.5 s; (**c**) 0.9 s.

The residence time of the particles in the renewable dielectric of canola oil inside the 4 mm hole within 1 s and their size distribution characteristics, during processing of a hole with a diameter of 4 mm, are selected to be demonstrated in Figure 4. From the figure, it can be observed that there is a significant difference in the motion behavior of particles of different sizes inside the holes, indicating that the particle size has an important effect on their migration characteristics. Specifically, large-sized particles are more likely to be expelled from the gap compared to small-sized particles. This is mainly due to the fact that large-sized particles have a larger mass and are subjected to more significant inertial and gravitational forces under hydrodynamic forces and therefore are more likely to move outward and be carried away from the gap during processing and to be discharged from the gap faster under the effect of the hydrodynamic force. In contrast, small-sized particles are more easily suspended in the gap between the electrode and the workpiece due to their relatively large surface area and low mass and momentum. This suspension property leads to a weaker migration of small-sized particles in the fluid medium, which cannot easily overcome the fluid viscous resistance to escape from the gap and thus remain in for a longer period of time. This effect of particle size on the migration behavior may further affect the stability of the EDM region, which in turn affects the machining efficiency and surface quality [29].

**Figure 5:**
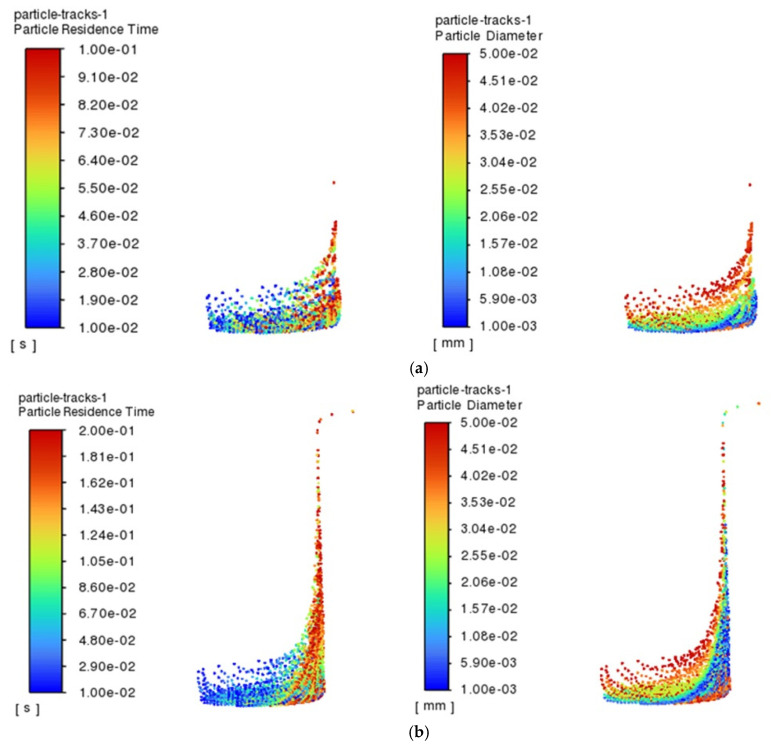

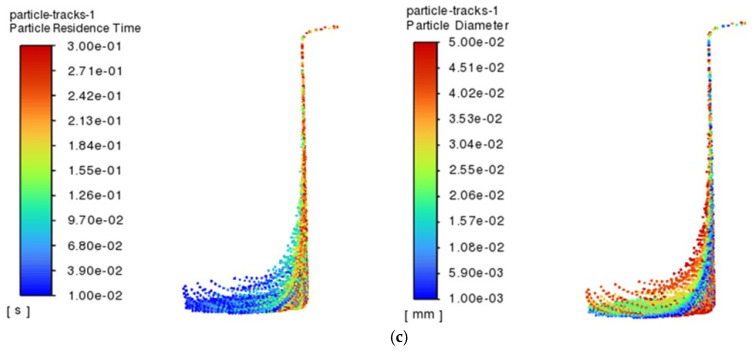
Residence time and size distribution of particles at 0.1~0.3 s for a diameter of 1 mm and a renewable dielectric of sunflower seed oil: (**a**) 0.1 s; (**b**) 0.2 s; (**c**) 0.3 s.

When processing a hole with a diameter of 1 mm, the escape rate of particles shows a trend of increasing and then stabilizing, and the escape rate of particles is at the lowest level at the beginning of processing, when most of the particles generated during processing are still retained in the hole because the hydrodynamic conditions have not yet been established completely. With the passage of time, the flow field inside the hole gradually evolves, and the escape rate gradually rises, reaching the highest value at 0.6 s, after which the trend tends to stabilize gradually. In order to deeply analyze the behavioral characteristics of the particles in the holes, the particles in the time period of 0.1~0.3 s are selected for the analysis, as depicted in Figure 5, which shows the residence time and size distribution of the particles in the canola oil renewable dielectrics in 1 mm holes in the time period of 0.1~0.3 s. The analysis results show that the average residence time of the particles is decreasing with the increase in time, and this phenomenon may be caused by the evolution of the flow field in the hole, which makes the particles escape more easily under the force. In addition, further analysis of the particle sizes as well as their residence times reveals that large-sized particles are the first to escape, while small-sized particles are more likely to be retained near the hole wall. Large particles, due to their larger mass, are able to break through the fluid resistance into the main flow zone faster under the effect of inertia, thus leaving the hole faster.

### 3.2. Effects of Dielectrics

**Figure 6:**
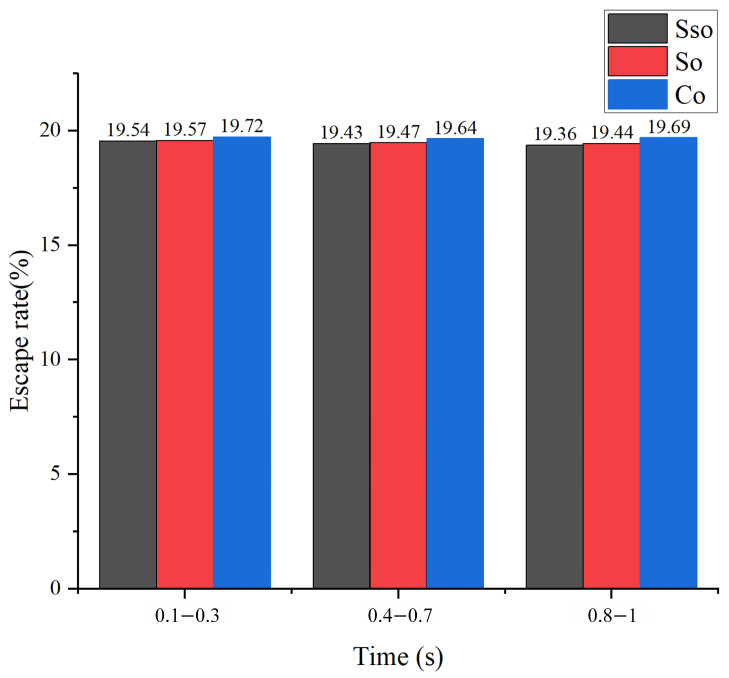
Comparison of average particle escape rates for different renewable dielectrics at different time periods for a hole with diameter of 4 mm.

The average escape rates of particles from different renewable dielectrics at different moments when processing 4 mm holes are shown in Figure 6, and the results indicated that the average escape rate of particles from the renewable dielectrics in canola oil is the highest in 1 s compared to other groups of experiments with the same processing diameter, with a value of 19.683%, which is higher than sunflower seed oil by 0.24% (than soybean oil by 0.19%). Among the three renewable dielectrics, namely sunflower seed oil, soybean oil, and canola oil, the viscosity of canola oil occupies the first place, which is about 1.5 times of that of soybean oil and 2.7 times of that of sunflower seed oil. The oils with high viscosity exhibited higher escape rates than the other oils in the same group.

**Figure 7:**
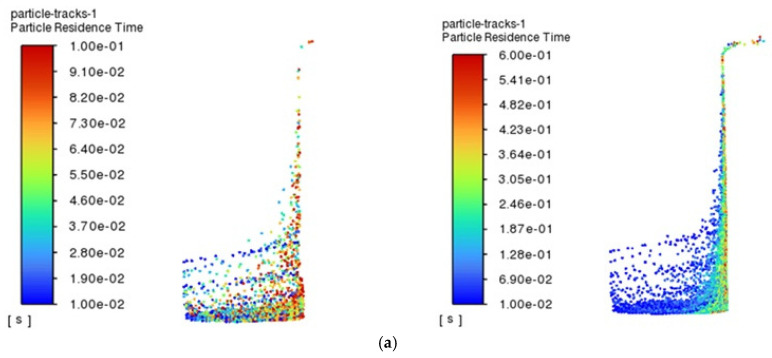

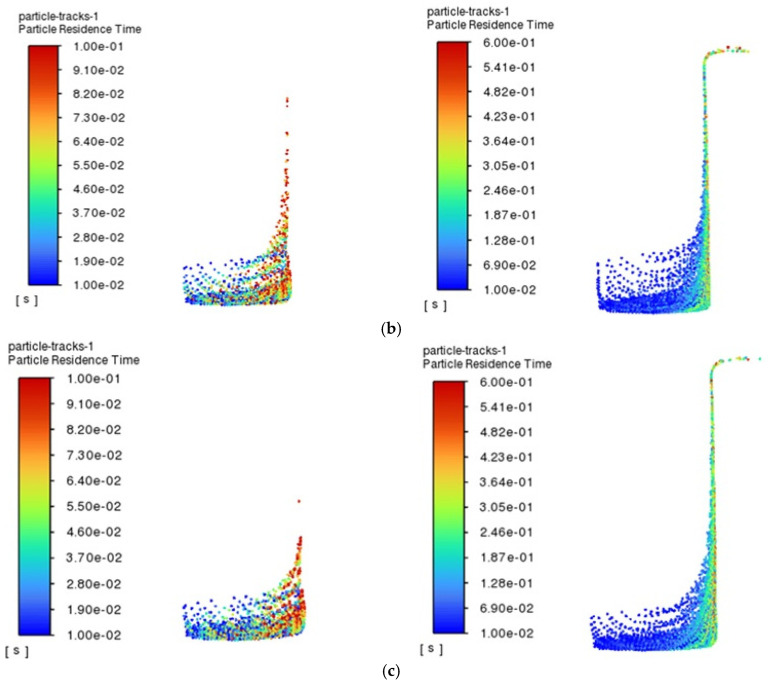
Residence time and size distribution of particles in different renewable dielectrics with diameters of 1 mm, 0.1 s, and 0.6 s: (**a**) sunflower seed oil; (**b**) soybean oil; (**c**) canola oil.

In contrast, when processing 1 mm holes, the viscosity of the oil have a significant effect on particle motion during EDM processing, with particles in the most viscous canola oil having the highest escape rate and the lowest in sunflower seed oil. Figure 7 provides a more intuitive visualization of the three regenerative dielectrics at 0.1 s and 0.6 s particle residence times. It is clearly shown that, in canola oil, the distribution of particles is denser, which is mainly due to the fact that the flow state of the oil with higher viscosity tends to be more laminar when it flows in the small holes. And the flow of the fluid in the laminar state is more regular compared to turbulence, which reduces the unfavorable effect of the complex-disturbed flow due to turbulence on the motion of the particles and makes the particles more likely to pass through the small holes along the direction of the flow of the fluid. This kind of stable flow environment helps to reduce the random movement of particles in the fluid, which in turn increases their escape rate. In addition, since oil with high viscosity exerts a stronger drag effect on the particles, it can effectively drive the particles to move with the fluid, making it easier for them to pass through the small holes.

Therefore, in a hole with a diameter of 4 mm, the processing area is relatively open compared to a hole with a diameter of 1 mm, and large particles have more chances to contact with the hole wall, and the viscous oil will form a stronger adhesion between the particles and the hole wall, so that the particles are more likely to be adsorbed or retained by the hole wall, preventing them from escaping. In small holes, the processing area is relatively small, and, under the action of high-viscosity oil, a stronger shear stress and pressure gradient are formed in the holes, especially under the action of thermal bubbles generated during the discharge; the local pressure fluctuation may be enhanced, which prompts the small particles to be carried out of the holes more easily, and at the same time, the dragging effect of the highly viscous oil on the particles is relatively more significant.

## 4. Experimental Results and Analysis

**Figure 8:**
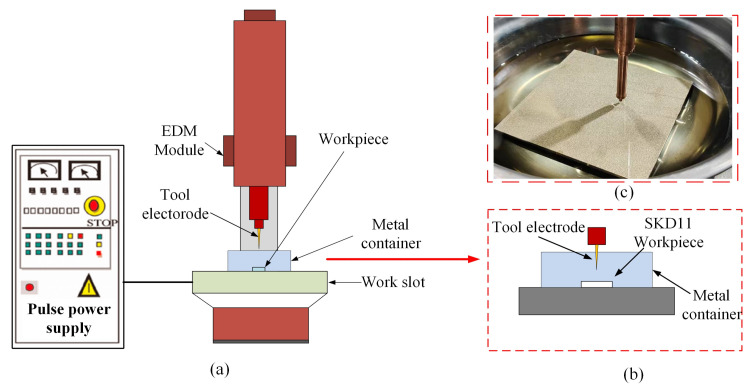
Schematic diagram of renewable dielectrics for drilling holes in mold steel SKD11; (**a**) EDM equipment; (**b**) workpiece attachment for machining; (**c**) SKD11 workpiece drilled by EDM.

In this experiment, a metal container was used to hold the renewable dielectrics, and the SKD11 to be processed was put into the metal container, and the scale of the SKD11 workpiece used for the experiment was 80 mm × 80 mm × 30 mm. The experimental setup is shown in Figure 8, in which the electrodes are shaped as a conical tool and made of copper material; the diameter (small end face) of this conical electrode is 1000 μm, and the taper difference is 1°. The main reason for choosing copper as the material for the tool electrode is its superior machining properties. Firstly, copper has a low-tool-wear ratio, which helps to increase the service life of the electrode. Secondly, the excellent electrical conductivity of the copper material facilitates stable energy transfer during the EDM process. Finally, in this study, optical microscopic measurements of the machining characteristics after EDM drilling were carried out using a Leica DVM6 super-depth-of-field microscope, which is characterized by a high resolution and large depth-of-field and is able to clearly capture the surface morphology and machining characteristics of the microstructures.

In order to study the processing performance in detail, this experiment adopted an orthogonality test design, i.e., a small number of experiments to examine the effect of process parameters to obtain the characteristics of high efficiency, uniformity, and reliability. Four processing parameters were selected for this experiment, namely, current, pulse width, pulse interval, and renewable dielectrics. The orthogonal test method L18(6,1,3,3) with one factor with six levels and three factors with three levels was used in the processing of SKD11 samples, and the experimental input process parameters and their levels are shown in Table 5, and the other input process parameters, such as server voltage and polarity, were kept constant, and a total of 18 microvia drilling experiments were carried out on the SKD11 samples.

### 4.1. Processing Performance Indicators

EDM stands out due to its ability to non-contact process materials with desired shapes through multi-pulse discharge. When investigating the machinability of EDM, several performance metrics are commonly used to evaluate the process machinability, including MRR, overcut, edge deviation, EEV, etc. These metrics are affected by the process parameters, which are mainly electrical (e.g., pulse current, pulse width, pulse spacing, and voltage) and non-electrical (e.g., electrode material, working fluid, and discharge gap) parameters.

Among them, overcut is an important geometric error metric mainly used to measure machining accuracy. It is defined as the difference between the drilling radius and the tool radius, i.e., the amount of additional material removed during machining due to the discharge gap and the extended effect of material erosion, which affects the amount of overcut due to a variety of factors, including the discharge energy, pulse gap, electrode material, working fluid characteristics, and workpiece material. It can be expressed in Equation (12),

(12) $$Overcut=\frac{\left(D_{w}-D_{t}\right)}{2}$$

where $D_{w}$ is the average diameter of the hole, which is calculated as the average of the diameters of the irregular internal tangent circles of the drill hole, and $D_{t}$ is the outer diameter of the tool.

The edge deviation of micro-holes is closely related to their surface finish, which is one of the important parameters for assessing the machining quality of micro-holes. The machining quality of micro-holes can be effectively assessed by analyzing the difference between the maximum and minimum diameters of the irregular edges of the micro-holes. Edge deviation is an important indicator for assessing the quality of hole machining, and a large edge deviation usually means that there is a large instability in the machining process. The edge deviation of the hole is accomplished by evaluating the edge of the micro-hole, which is determined by Equation (13),

(13) $$Edge\;deviation=\frac{\left(D_{\max}-D_{\min}\right)}{2}$$

where, $D_{max}$ is the diameter of the internal tangent circle with maximum irregularity, and $D_{min}$ is the diameter of the internal tangent circle with minimum irregularity.

MRR is an important parameter for measuring the efficiency of the micro-hole drilling process and has a wide range of applications in precision manufacturing. Typically, MRR is defined as the volume of material removed from the workpiece per unit time, usually in cubic millimeters per minute (mm^3^/min). It is not only one of the key indicators for evaluating the productivity of the machining process, but also has a direct impact on the machining efficiency, cost control, and the quality of the final product. The calculation of MRR can be expressed as Equation (14),

(14) $$MRR=\frac{V}{t}=\frac{\pi D_{w}^{2}h}{4t}$$

where *V* is the volume of material removed, *h* is the depth of cut of the hole, and *t* is the machining time.

In small-hole drilling, the MRR is influenced by a number of factors, such as material properties, machining parameters, and the characteristics of the working fluid. Higher MRR usually means faster machining speeds, which can lead to increased productivity, but, if MRR is increased too much, it may lead to reduced hole quality or even workpiece damage.

EEV is defined as the input energy (J/mm^3^) required to remove 1 mm^3^ of workpiece. This parameter is used during machining to assess the efficiency of energy utilization and to measure the effect of different machining methods or process parameters on energy consumption. Equation (15) provides the definition of EEV,

(15) $$EEV=\frac{P}{MRR}=\frac{UI\eta \times 60/1000}{\frac{\pi D_{w}^{2}h}{4t}}=\frac{6UI\eta t}{25\pi D_{w}^{2}h}$$

where *U* is the discharge voltage, *I* is the discharge current, and η is the pulse duty cycle.

### 4.2. Experimental Results

**Figure 9:**
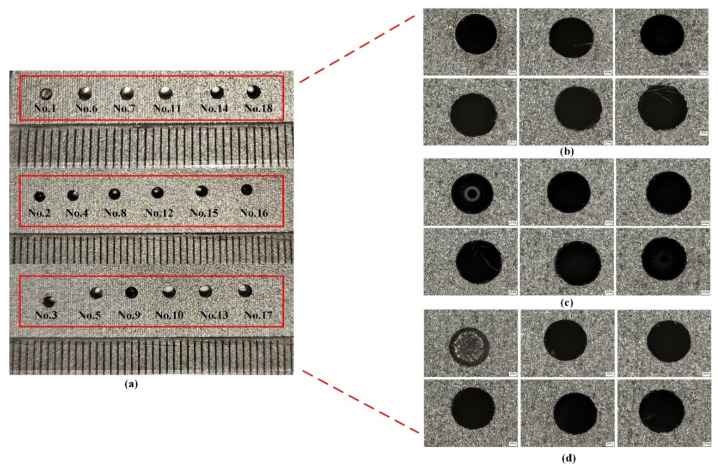
Small holes machined arrays in different dielectrics and their local magnification; (**a**) small holes arrays; (**b**) sunflower seed oil; (**c**) canola oil; (**d**) soybean oil.

In order to study the machinability of drilling micro-holes in SKD11 using renewable dielectrics in electric discharge machining, the machining performance was experimentally investigated under a different current, pulse width, and pulse interval. Given that this study employed industrial-grade high-precision experimental equipment, the obtained data possess high accuracy and reliability. Meanwhile, considering the substantial processing costs associated with each experimental condition, only a single experiment was conducted for each test scenario. The machined experimental samples are shown in Figure 9. To ensure the accuracy of the data, eight diameter measurements were taken for each hole machined after the experiments using an ultra-depth-of-field microscope to collect the measured data, and the values of the machinability performance are shown in Table 6, where the values of the sunflower seed oil renewable dielectric, canola oil renewable dielectric, and soybean oil renewable dielectric are denoted by numbers 1, 2, and 3, respectively. The EDM input process parameters such as current, pulse width, pulse interval, and dielectric are selected to study the effect of these parameters on MRR, overcut, edge deviation, and EEV.

**Figure 10:**
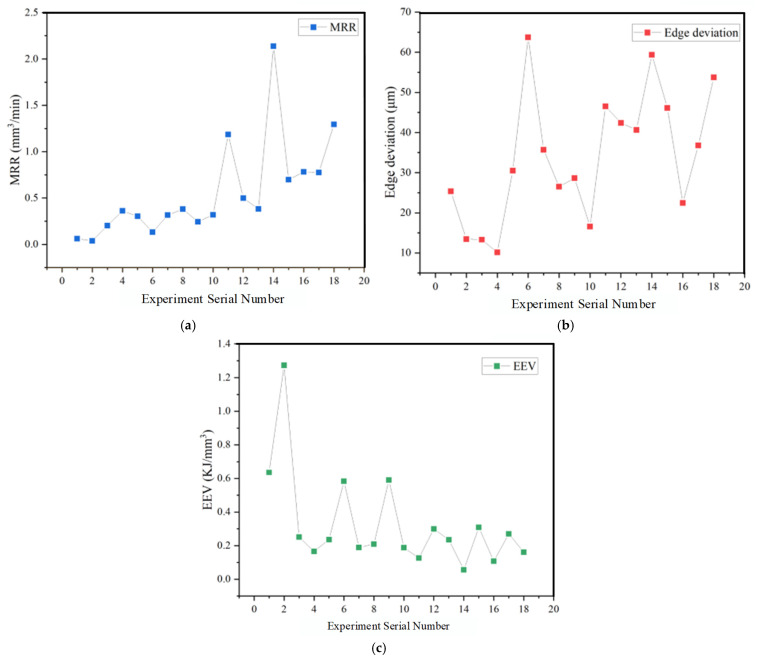
Results of EDM SKD 11; (**a**) MRR, (**b**) Edge deviation, (**c**) EEV.

Figure 10 visualizes the MRR, edge deviation, and EEV for 18 sets of experiments using EDM for machining SKD11 in renewable dielectrics. The best value of MRR of 2.137 mm^3^/min is achieved for the 14th set of experiments with a current of 6 A, a pulse width of 200 μs, a pulse interval of 300 μs, and a renewable dielectric of sunflower seed oil, which is much higher than the results of the other sets of experiments. The best value of 10.131 μm for marginal deviation is obtained in the group 4 experiment with a current of 2 A, a pulse width of 100 μs, a pulse interval of 200 μs, and a renewable dielectric of canola oil, and the best value of 0.05615 KJ/mm3 for EEV is obtained in the group 14 experiment.

### 4.3. Effect of Process Parameters on MRR

**Figure 11:**
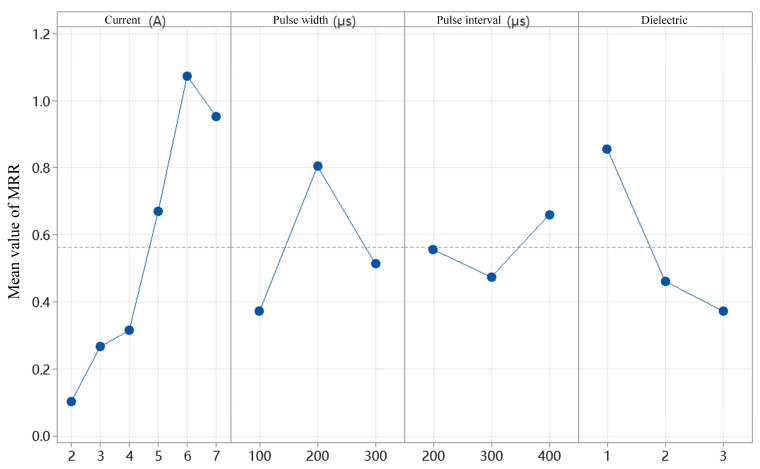
Main effect plot of mean MRR under different currents, pulse durations, pulse intervals, and dielectric fluid conditions.

MRR refers to the volume of material removed per unit time. A higher MRR indicates greater production efficiency; thus, it is considered a critical technical indicator in manufacturing processes [30]. To determine the significance of variations, the mean MRR values under different currents, pulse durations, pulse intervals, and dielectric fluids are calculated, as shown in Figure 11. The maximum MRR of 2.137 mm^3^/min is achieved at a current of 6 A, a pulse duration of 200 μs, a pulse interval of 400 μs, and when using sunflower seed oil as the renewable dielectric. Conversely, the minimum MRR of 0.038 mm^3^/min occurs under a current of 2 A, a pulse duration of 200 μs, a pulse interval of 300 μs, and when using canola oil as the dielectric. It can also be observed that MRR generally increases with increasing current and then decreases after a certain point. Overall, MRR tends to rise with higher discharge currents. The highest average MRR of approximately 1.09 mm^3^/min is obtained at 6 A, while the average MRR at 2 A is only 0.11 mm^3^/min, representing a 9.9-fold increase—indicating a significant effect. As the pulse duration increased, MRR showed a trend of first increasing and then decreasing, with the optimal value appearing at 200 μs. In contrast, MRR exhibited an inverse relationship with the pulse interval, suggesting that either excessively long or short pulse durations and intervals are detrimental to material removal. The use of sunflower seed oil as a renewable dielectric resulted in the highest average MRR of 0.92 mm^3^/min, which can be attributed to its relatively low viscosity compared to the other tested oils. Although lower-viscosity dielectrics typically have weaker debris flushing capabilities during small-hole machining, they can enhance micro-EDM performance through mechanisms such as stable discharge channel formation, rapid cooling, and improved high-frequency pulse response. Table 7 presents the mean response values of MRR under different combinations of current, pulse duration, pulse interval, and dielectric fluid. The results indicate that current and dielectric type exert the most significant influence on MRR.

**Figure 12:**
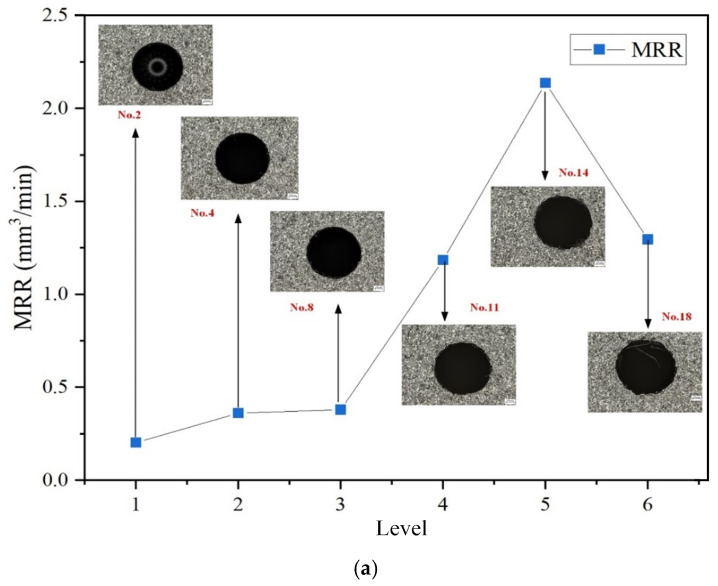

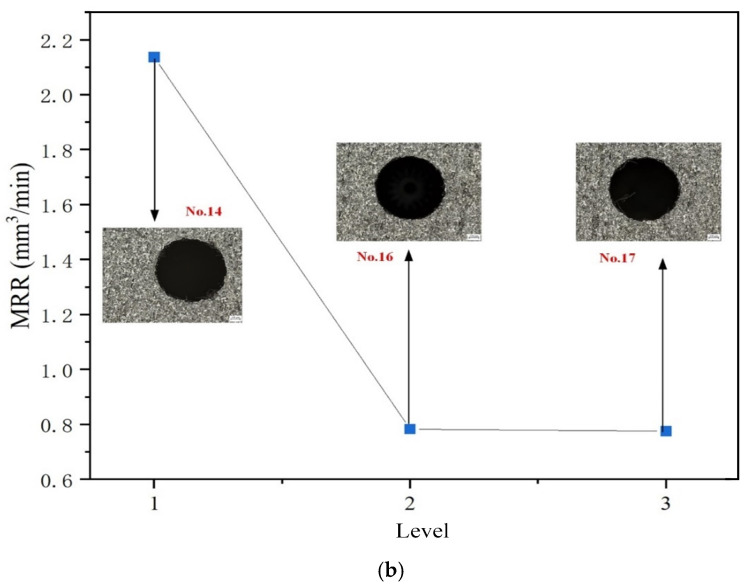
Influence of current and dielectric fluid on MRR; (**a**) Current; (**b**) Dielectric fluid.

Figure 12 illustrates the optimal MRR performance under the two most influential factors—current and dielectric fluid—as determined by the ranking analysis. Figure 12a shows the optimal MRR performance across different current levels. The best MRR value is obtained at a current of 6 A, which can be attributed to the increased energy released during pulse discharge with the higher current. Under the effect of instantaneous high temperature, the surface material of the workpiece rapidly melts or even vaporizes. Meanwhile, the dielectric fluid in the discharge channel rapidly vaporizes and expands due to the heat, exerting an impact force on the molten material on the workpiece surface, thereby accelerating the material removal process. Figure 12b displays the optimal MRR performance across different dielectric fluids. The highest MRR value is achieved when sunflower seed oil, a renewable dielectric, is used for EDM drilling. In contrast, the average MRR values obtained with canola oil and soybean oil are comparatively lower and showed little difference between them. Among the six current levels, the top three performing conditions in terms of MRR all use sunflower seed oil as the dielectric. Furthermore, in the experiments that yielded the best results, a pulse duration of 200 μs is selected in half of the cases. From the perspective of both current and dielectric fluid, Experiment No. 14 achieved the highest MRR, with an input current of 6 A, a pulse duration of 200 μs, and the use of sunflower seed oil as the dielectric. This indicates that employing a high current, short pulse interval, and a low-viscosity dielectric fluid is more conducive to effective material removal.

### 4.4. Influence of Process Parameters on Edge Deviation

**Figure 13:**
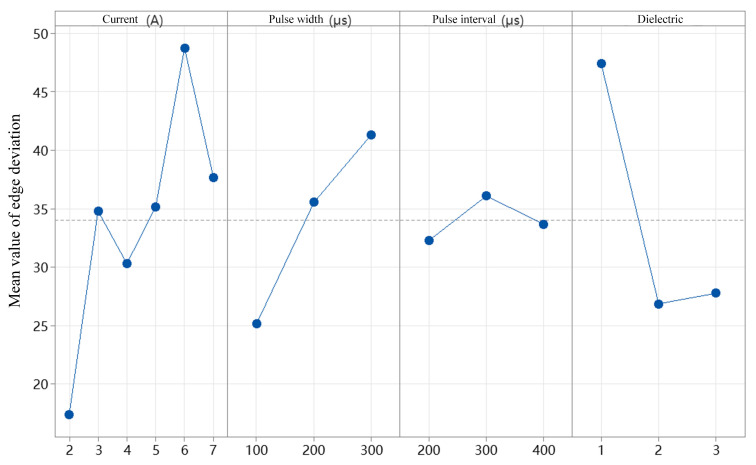
Main effect plot of mean edge deviation under different currents, pulse durations, pulse intervals, and dielectric fluid conditions.

Figure 13 shows the mean edge deviation under different levels of current, pulse duration, pulse interval, and dielectric fluid. As visually indicated in the figure, the variations in edge deviation are more pronounced under the factors of current and dielectric fluid. With the increase in current, the edge deviation first increases, then decreases, and subsequently increases again, forming a fluctuating trend. When the current is 2 A, the mean edge deviation is 17.369 μm, while at 6 A, it reaches 48.744 μm, which is approximately 2.8 times greater. Regardless of how much the current increases, the edge deviation remains higher than that at 2 A. This is because a higher current results in greater discharge energy, which enhances material removal but can also lead to reduced stability in the removal process, causing localized over-erosion or under-erosion. As the pulse duration increases, the mean edge deviation shows a consistent rising trend. This is due to the increased discharge energy per unit time, which removes more material per pulse. Consequently, the concentration of debris particles in the discharge gap increases, interfering with the stability of the discharge channels and leading to localized discharge shifts and edge deviation. Among the dielectric fluids tested, sunflower seed oil resulted in the highest mean edge deviation, while canola oil yielded the lowest. As demonstrated in the simulation experiments in Section 3, during EDM drilling of 1 mm diameter holes, higher-viscosity dielectrics more effectively flush away erosion by-products from the machining area, improving discharge stability. This in turn leads to smaller edge deviation and better machining precision. Table 8 presents the mean response values of edge deviation under various levels of current, pulse duration, pulse interval, and dielectric fluid. Among these factors, current and dielectric fluid have the most significant influence on edge deviation. Therefore, these two factors are selected for further analysis.

**Figure 14:**
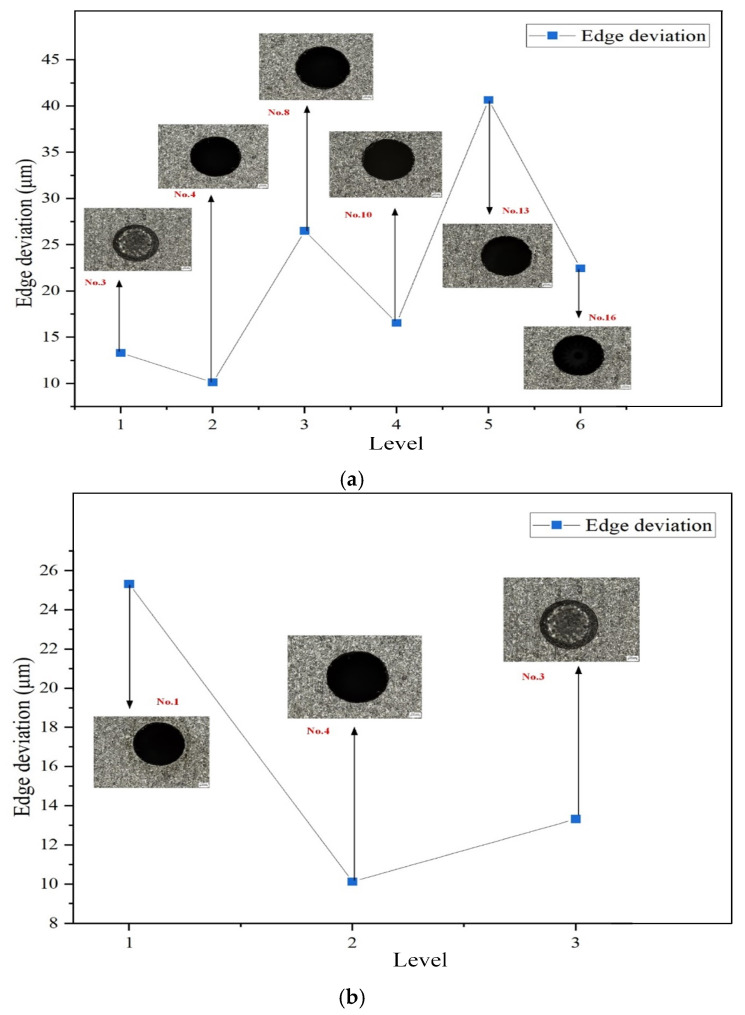
Effects of discharge current and dielectric fluid on edge deviation: (**a**) discharge current; (**b**) dielectric fluid.

A smaller edge deviation indicates higher machining quality. Figure 14a,b illustrate the influence of the discharge current and dielectric fluid, respectively, on edge deviation in small-hole EDM. First, from the perspective of discharge current, lower current levels result in reduced edge deviation. When the current is set to 3 A, the edge deviation of the machined holes is minimized, indicating improved machining performance. A lower current ensures a more stable discharge process, thereby enhancing machining quality. This is primarily because a reduced discharge current leads to smaller energy fluctuations, stabilizing the process and mitigating issues such as excessive erosion or non-uniform localized discharges caused by high currents. Secondly, regarding the dielectric fluid, as shown in Figure 14b, when canola oil is used as the renewable dielectric, the edge deviation is further reduced. This improvement is mainly attributed to the relatively high viscosity of canola oil, which exceeds that of the other two dielectrics considered. In small-hole EDM, a high-viscosity dielectric not only enhances debris removal efficiency but also effectively suppresses random arc deflection, leading to more uniform and controllable discharges. Consequently, this reduces material removal non-uniformity and machining errors. Moreover, higher viscosity improves the dielectric’s cooling capacity, thereby limiting the extension of the heat-affected zone and further optimizing the overall machining quality.

### 4.5. Effect of Process Parameters on EEV

**Figure 15:**
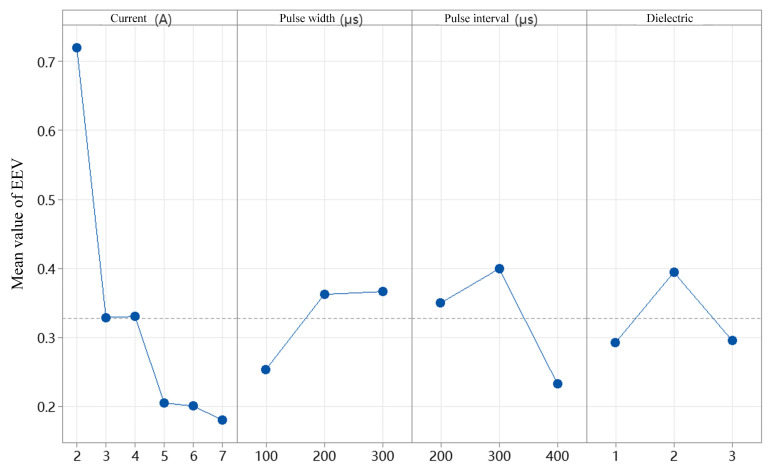
Main effect plots of average EEV under different discharge currents, pulse durations, pulse intervals, and dielectric fluids.

The EEV refers to the amount of energy consumed to remove a unit volume of material in EDM. A lower EEV indicates reduced energy consumption, aligning more closely with the principles of green manufacturing. Figure 15 presents the mean EEV values under various conditions, including different discharge currents, pulse durations, pulse intervals, and dielectric fluids. It is evident from the figure that the discharge current exhibits an inverse relationship with EEV: as the current increases, EEV decreases. Specifically, when the current increases from 2 A to 7 A, the average EEV drops from 0.73 kJ/mm^3^ to 0.18 kJ/mm^3^, with the former being approximately four times higher than the latter. In contrast, pulse duration shows a direct relationship with EEV; as the pulse duration increases, EEV increases accordingly. When the pulse duration rises from 100 μs to 300 μs, the average EEV increases from 0.25 kJ/mm^3^ to 0.37 kJ/mm^3^. With respect to pulse interval, the mean EEV initially increases and then decreases as the interval lengthens, reaching an optimal value at a pulse interval of 400 μs. Regarding the dielectric fluid, the average EEV values obtained using sunflower seed oil and soybean oil as renewable dielectrics are comparable. The viscosities of sunflower seed oil and soybean oil are 0.0049 and 0.0085, respectively, both lower than that of canola oil (0.013), which may explain the similar energy efficiency observed. Table 9 summarizes the mean response of EEV under different currents, pulse durations, pulse intervals, and dielectric fluids. Among these parameters, discharge current and pulse interval exert the most significant influence on EEV. Therefore, these two parameters were selected for further experimental analysis.

**Figure 16:**
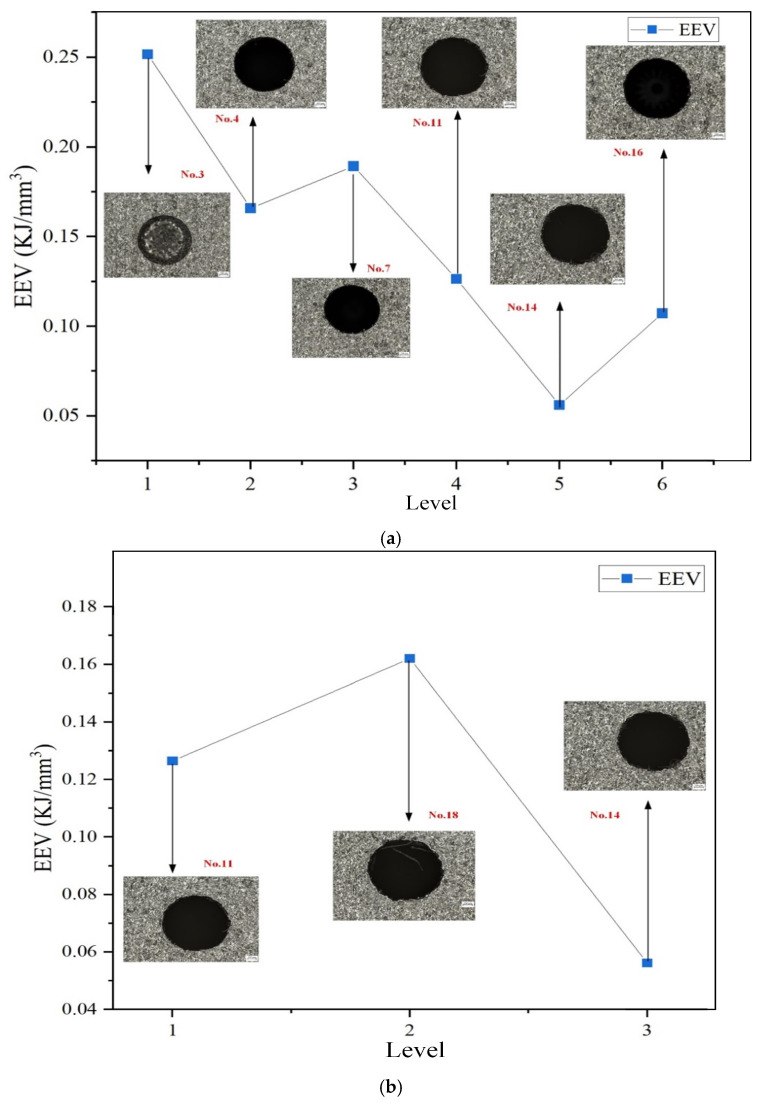
Effects of discharge current and pulse interval on edge deviation: (**a**) discharge current; (**b**) pulse interval.

A lower EEV indicates that less energy is required to remove the same volume of material, implying higher machining efficiency. Figure 16 illustrates the influence of discharge current and pulse duration on EEV in EDM hole machining. The EEV value generally decreases with increasing current, signifying improved efficiency. The optimal EEV is achieved at a discharge current of 6 A, where the lowest energy consumption per unit volume is observed.

## 5. Conclusions

This paper investigates the EDM process of small holes in renewable dielectric materials through simulation and experimentation. Fluent was used to perform transient simulations of EDM using the DPM method, and a single-factor experimental method was employed to study the effects of hole diameter and three types of renewable dielectric materials on performance. Furthermore, using orthogonal experimental design, the machining performance of SKD11 die steel under different current, pulse width, pulse interval, and renewable dielectric conditions was compared and analyzed, including MRR, edge deviation, and EEV. The relevant research conclusions are as follows:

(1) This study revealed the quantitative relationship between hole diameter and particle escape rate and identified the advantageous mechanism of high-viscosity media in small hole machining, providing theoretical guidance for process optimization in micro-hole EDM. The escape rate of metal particles in a 4 mm diameter hole is approximately 9.5 times that in a 1 mm diameter hole. Larger hole diameters facilitate the easier escape of metal particles. When hole diameters are relatively small, using a higher-viscosity oil as the regenerative dielectric results in better escape rates of erosion products within the hole.

(2) This study explains the advantageous mechanism of low-viscosity plant oils in micro-hole processing from the perspective of flow field simulation. The MRR value initially increases with increasing current and then begins to decrease after reaching a maximum value, indicating that the discharge energy should not be too high, as this can lead to large debris, thereby affecting discharge stability. The MRR value decreases with increasing viscosity; the MRR of sunflower seed oil as a regenerative dielectric is superior, reaching 0.8552 mm^3^/min, significantly higher than rapeseed oil at 0.4603 mm^3^/min and soybean oil at 0.3718 mm^3^/min.

(3) This study found that edge deviation is inversely proportional to MRR, with the lowest edge deviation of 10.131 μm achieved under low current and high-viscosity medium combinations. This further reveals the intrinsic mechanism linking viscosity and precision: high-viscosity media form laminar flow in small holes, reducing discharge instability. Additionally, precision improvements were achieved through medium selection optimization, providing a new approach for single-medium optimization.

(4) The EEV evaluation system established in this study shows that EEV decreases by approximately four times at 6 A compared to 2 A, filling a gap in green manufacturing evaluation metrics. Combined with traditional EDM environmental hazard research, the energy consumption analysis in this study lays the foundation for a full, life-cycle, environmental benefit assessment of plant-oil EDM.

## Figures and Tables

**Table 1:** Settings for experimental levels.

Parameter	Level	Value
Dielectric	4	Sunflower seed oil, soybean oil, and canola oil
Diameter of the hole (mm)	2	1, 4
Current (A)	1	2

**Table 2:** Comparison of physical properties of kerosene, sunflower seed oil, soybean oil, and canola oil.

Dielectric	Viscosity (mPa·s)	Flash Point (°C)	Ignition Point (°C)	Density (g/cm^3^)
Kerosene	0.0024	52	80~84	0.8000
Sunflower seed oil	0.0049	250	335	0.9200
Soybean oil	0.0085	254	351	0.9263
Canola oil	0.0130	246	240	0.9115

**Table 3:** Parameter settings for the proposed simulation model.

Project	Content
Material	Steel
Injection Velocity	4 m/s
Particle Concentration	1.46 × 10^−16^ kg/s
Max Diameter	0.05 mm
Min Diameter	0.001 mm
Mean Diameter	0.03 mm

**Table 4:** Simulation experiment results.

No.	Diameter (mm)	Medium	Escape Rate at Different Time (%)									
			0.1 s	0.2 s	0.3 s	0.4 s	0.5 s	0.6 s	0.7 s	0.8 s	0.9 s	1 s
1	1	Sso	0.70	0.970	1.62	1.94	2.30	2.45	2.43	2.37	2.32	2.26
2	1	So	0.16	0.77	1.70	2.16	2.66	2.90	2.96	2.97	2.96	2.94
3	1	Co	0	0.61	1.78	2.29	2.83	3.01	3.04	3.03	3.03	3.05
4	4	Sso	19.71	19.50	19.42	19.46	19.40	19.45	19.39	19.39	19.47	19.23
5	4	So	19.68	19.51	19.53	19.55	19.36	19.40	19.55	19.37	19.41	19.55
6	4	Co	19.96	19.53	19.68	19.62	19.78	19.48	19.67	19.70	19.76	19.61

**Table 5:** Experimental input process parameters and their levels.

Parameter	Level	Value
Current (A)	6	2, 3, 4, 5, 6, 7
Pulse width (μs)	3	100, 200, 300
Pulse interval (μs)	3	200, 300, 400
Dielectric	3	1-sunflower seed oil, 2-canola oil, 3-soybean oil

**Table 6:** SKD11 small hole machining performance under different input process parameters.

Experiment Number	Process Parameters				Machining Performance		
	Current (A)	Pulse Width (μs)	Pulse Interval (μs)	Dielectric	MRR mm^3^/min	Edge Deviation μm	EEV KJ/mm^3^
1	2	100	200	1	0.063	25.324	0.63589
2	2	200	300	2	0.038	13.459	1.27278
3	2	300	400	3	0.204	13.324	0.25166
4	3	100	200	2	0.362	10.131	0.16582
5	3	200	300	3	0.304	30.506	0.23655
6	3	300	400	1	0.132	63.730	0.58368
7	4	100	300	1	0.317	35.689	0.18931
8	4	200	400	2	0.381	26.512	0.21013
9	4	300	200	3	0.244	28.657	0.59112
10	5	100	400	3	0.320	16.573	0.18773
11	5	200	200	1	1.186	46.533	0.12647
12	5	300	300	2	0.500	42.396	0.30006
13	6	100	300	3	0.383	40.673	0.23507
14	6	200	400	1	2.137	59.386	0.05615
15	6	300	200	2	0.698	46.142	0.30927
16	7	100	400	2	0.783	22.470	0.10731
17	7	200	200	3	0.776	36.794	0.27049
18	7	300	300	1	1.296	53.709	0.16205

**Table 7:** Mean response table of MRR with respect to current, pulse duration, pulse interval, and dielectric fluid.

Level	Current (A)	Pulse Duration (μs)	Pulse Interval (μs)	Dielectric Fluid
1	0.1017	0.3713	0.5548	0.8552
2	0.2260	0.8037	0.4730	0.4603
3	0.3140	0.5123	0.6595	0.3718
4	0.6687			
5	1.0727			
6	0.9517			
Delta	0.9710	0.4323	0.1865	0.4833
Rank	1	3	4	2

**Table 8:** Mean response table of edge deviation with respect to current, pulse duration, pulse interval, and dielectric fluid.

Level	Current (A)	Pulse Duration (μs)	Pulse Interval (μs)	Dielectric Fluid
1	17.37	25.14	32.26	47.40
2	34.79	35.53	36.07	26.85
3	30.29	41.33	33.67	27.75
4	35.17			
5	48.73			
6	37.66			
Delta	31.36	16.18	3.81	20.54
Rank	1	3	4	2

**Table 9:** Mean response table of EEV with respect to discharge current, pulse duration, pulse interval, and dielectric fluid.

Level	Current (A)	Pulse Duration (μs)	Pulse Interval (μs)	Dielectric Fluid
1	0.7201	0.2535	0.3498	0.2923
2	0.3287	0.3621	0.3993	0.3942
3	0.3302	0.3663	0.2328	0.2954
4	0.2048			
5	0.2002			
6	0.1800			
Delta	0.5402	0.1128	0.1665	0.1020
Rank	1	3	2	4

## Data Availability

Data is available when request.
